# Strength training for arterial hypertension treatment: a systematic review and meta-analysis of randomized clinical trials

**DOI:** 10.1038/s41598-022-26583-3

**Published:** 2023-01-05

**Authors:** Rafael Ribeiro Correia, Allice Santos Cruz Veras, William Rodrigues Tebar, Jéssica Costa Rufino, Victor Rogério Garcia Batista, Giovana Rampazzo Teixeira

**Affiliations:** 1 Department of Physical Education, School of Technology and Sciences, São Paulo State University-UNESP, Street Roberto Simonsen, 305, Presidente Prudente, SP 19060-900 Brazil; 2 Multicenter Graduate Program in Physiological Sciences, SBFis, São Paulo State University (UNESP), Araçatuba, São Paulo Brazil; 3 Center of Clinical and Epidemiological Research, University Hospital, University of São Paulo-USP, Sao Paulo, Brazil

**Keywords:** Cardiovascular biology, Cardiovascular biology, Metabolism

## Abstract

Cardiovascular diseases are the leading cause of death in the world and arterial hypertension (AH) accounts for 13.8% of deaths caused by cardiovascular diseases. Strength training interventions could be an important alternative tool for blood pressure control, however, consistent evidence and the most effective training protocol for this purpose are yet to be established. The current study used the Cochrane methodology to systematically review randomized controlled trials (RCTs) that investigated the effect of strength training on blood pressure in hypertensive patients. A systematic search was conducted in the PubMed, EMBASE, Scopus, Cochrane Library, and World Health Organization databases. This review included controlled trials that evaluated the effect of strength training for 8 weeks or more in adults with arterial hypertension, published up to December 2020. Data are described and reported as the weighted mean difference of systolic and diastolic pressure and a 95% confidence interval. Protocol registration: PROSPERO registration number CRD42020151269. A total of 14 studies were identified, including a combined total of 253 participants with hypertension. The meta-analysis showed that mean values of systolic blood pressure (SBP) and diastolic blood pressure (DBP) decreased significantly after strength training interventions. The strongest effect of strength training on decreasing blood pressure was observed in protocols with a moderate to vigorous load intensity (> 60% of one-repetition maximum-1RM), a frequency of at least 2 times per week, and a minimum duration of 8 weeks. We concluded that strength training interventions can be used as a non-drug treatment for arterial hypertension, as they promote significant decreases in blood pressure.

## Introduction

Systemic arterial hypertension (SAH) is defined as increased and/or sustained systolic blood pressure levels above 140 mmHg and/or diastolic pressure above 90 mmHg^1^. Hypertension is one of the leading causes of death from cardiovascular diseases and affects approximately 1 billion people worldwide^2,3^. Systemic arterial hypertension is a multifactorial disease and can be triggered by factors such as physical inactivity, the intake of sodium-rich foods, obesity, alcohol, and tobacco consumption^4,5^. The non-pharmacological effect of physical exercise has the potential to facilitate hemodynamic changes, increased production of nitric oxide (NO), and changes in peripheral arterial resistance^6^. Current guidelines recommend the practice of physical exercise as part of primary and secondary prevention of cardiovascular diseases^7–9^. However, little has been discussed about the dose–response effects of strength training in the prevention and treatment of arterial hypertension.

Physical exercise improves and maintains health and reduces the risk of chronic diseases in healthy adults, as physical inactivity is considered one of the largest risk factors for chronic diseases. High rates of physical inactivity are associated with people with chronic diseases, demonstrating that physical activity programs and nutritional monitoring are required for these individuals, as part of disease prevention^10^. Acute exercise training responses generally promote increased heart rate (HR), increased blood vessel lumen (vasodilation) from increased (NO) synthesis, increased blood flow^11^, increased uptake of energy substrates^12^, and increased body temperature^13^. Long-term chronic physical training responses promote adaptations such as a decrease in resting HR, concomitant lowering of blood pressure (BP)^14,15^, improved heart efficiency^16^, and increased maximum oxygen volume (VO_2_máx)^17^. Systematic responses directly related to physical exercise depend on the load intensity, duration, and frequency at which it is performed^18^.

Recent research indicates that strength training has therapeutic potential against arterial hypertension^19,20^, however, the dose–response of strength training to high blood pressure is still unclear. This study aims to systematically examine long-term randomized clinical trials (RCTs) with the application of intervention protocols, to aid the development of a more effective prescription of training for diverse populations with arterial hypertension. We hypothesized that the effects of strength training could be associated with variables that make up the training volume and the intensity of performance in hypertensive people.

Therefore, the current systematic review aims to analyze the breadth of evidence on the treatment potential of strength training in adults and older people with hypertension, as well as to verify which load intensity and volume have the greatest effects. Previous reviews on the topic verified the effect of strength training on blood pressure^9,21^. However, the current work, in addition to being novel, provides additional evidence on the effect of training variables, such as load intensity, volume, weekly frequency, and age of the individuals.

## Results

### Study characteristics

**Figure 1:**
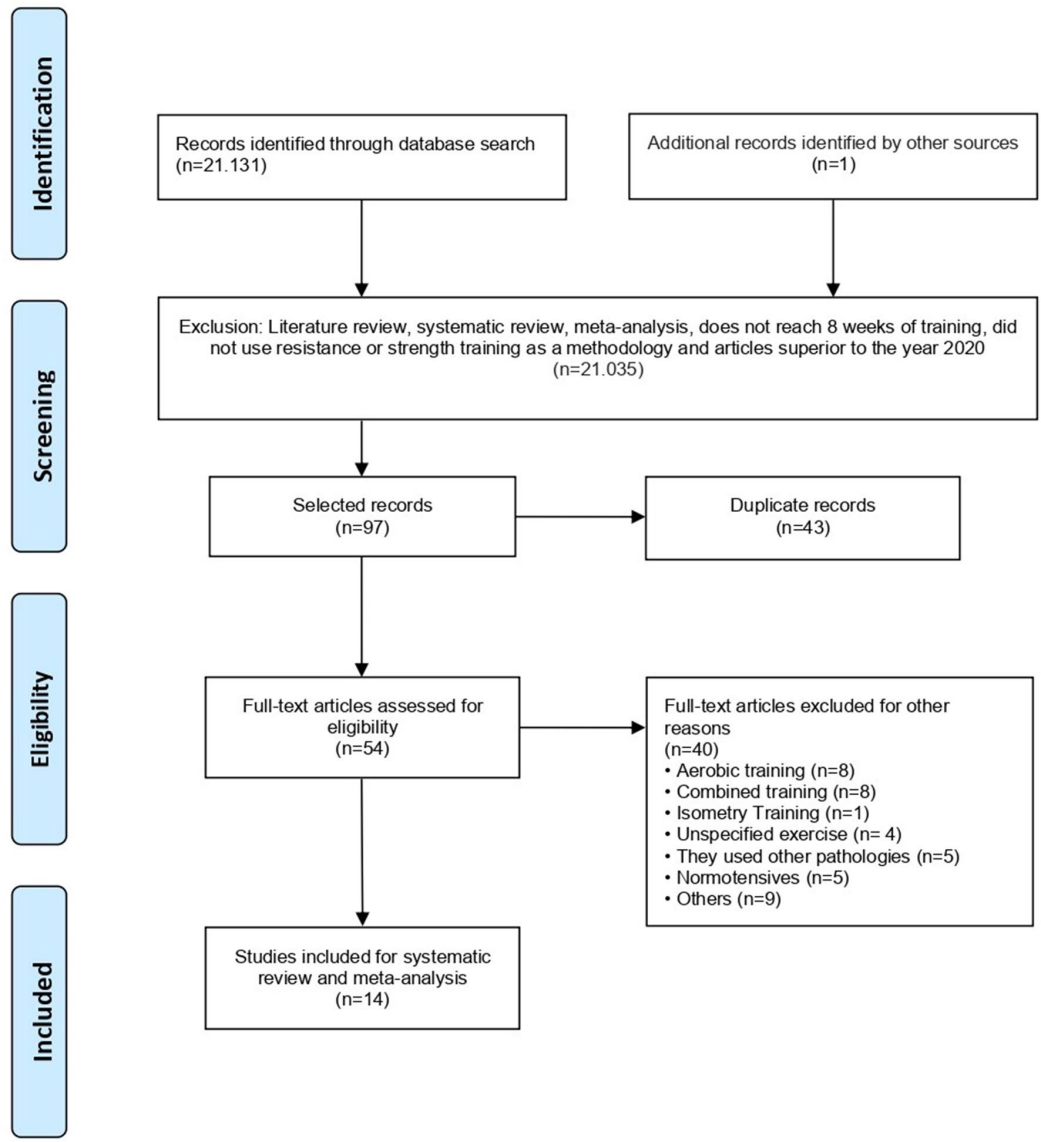
Flowchart corresponding to the identification, screening, eligibility, and included criteria of this study.

Figure 1 shows the number of trials included in the analysis. As a result of the investigation, we analyzed a total of 21,132 articles, of which 21,035 were excluded because they did not address the objective of the study including; systematic reviews and/or meta-analyses, those that did not reach the eight weeks of intervention, did not use resistance training or strength training as a work methodology, and articles published prior to 2009. After a more detailed analysis of the remaining 97 articles, 43 were excluded due to duplication, leaving 54 articles for full-text analysis. Of these, 40 articles did not meet the proposed objective or the eligibility and risk of bias criteria, and, thus, 14 articles that met the proposed criteria and were considered potentially relevant, were included and analyzed in the present systematic review.

For this review, all types of control groups were included, such as normotensive individuals^6,20^ and aerobic training^22,23^, but only the values of the hypertensive group were considered for the analysis, which represented a combined total of 253 participants, with a mean age of 59.66 years. To standardize the control groups across all studies, the control group analyzed in the meta-analysis was the hypertensive group that performed the training at baseline (called "before strength training" in the figures), and the experimental group was the same, but at the end of training (called "after strength training" in the figures).

### Data from the studies

The data on the description and characteristics of eligible studies are presented in detail in Table 1. The total sample size was 253 participants. The mean age was 59.66 years, and the populations of most studies were aged between 60 and 68 years, with only two studies including a younger population aged between 18 and 46 years. In this review, 14 studies were analyzed and included in the meta-analysis. Seven studies included patients of both sexes^22–28^, in another seven studies, the sample consisted of only females^6,20,29–32^, and one study included only a male sample^33^. Of the total sample, 75% were hypertensive, and in 11 of the 14 studies analyzed, the participants used anti-hypertensive drugs^6,20,22–25,27,29–32^, such as β-blocker, diuretics, calcium channel blockers, and angiotensin-converting enzyme inhibitors.

**Table 1:** Data referring to the total of individuals, age, average blood pressure, intervention data, and use of drugs of study.

Study	Total participants	Age (mean ± SD)	Sex	Drugs use	Physical exercise load intensity (intervention)	Weekly frequency (days)	Duration (weeks)	Baseline systolic blood pressure (mean ± SD)	Final systolic blood pressure (mean ± SD)	Δ %	Baseline diastolic blood pressure (mean ± SD)	Final diastolic blood pressure (mean ± SD)	Δ %
Beck, 2013^26^	15	21.1 ± 0.6	M/F	Unspecified	60% of 1RM	3	8	114.0 ± 2	104 ± 2	− 9.62	80 ± 2	73 ± 2	− 9.59
Brand, 2013^27^	8	53 ± 3	M /F	Yes (unspecified)	11–15 on Borg scale	3	48	130 ± 12	126 ± 10	− 3.17	86 ± 10	81 ± 9	− 6.17
Carvalho, 2013^22^	45	65.3 ± 3.4	M /F	Yes (Diuretics, and Ang II receptor antagonists)	50% of maximum heart rate	3	12	128.2 ± 4.0	126.3 ± 4.2	− 1.50	76.8 ± 4.1	76.6 ± 3.22	− 0.26
Carvalho, 2019^24^	5	60 ± 8	M /F	Yes (Beta-blocker)	11–13 on Borg Scale	3	12	121 ± 5	121 ± 12	0.00	69 ± 7	69 ± 7	0.00
Cunha, 2012^30^	9	69.1 ± 5.7	F	Yes (beta-blocker; ACEI; diuretic; calcium channel inhibitor)	2 series of eight repetitions with 8RM charge	3	8	126.9 ± 12.7	115.3 ± 22	− 10.06	68.1 ± 11.3	55.6 ± 5.5	− 22.48
Damorim, 2017^25^	28	62.8 ± 1.2	M /F	Yes (ACE inhibitors, diuretics, Ang II antagonists, calcium channel inhibitor)	60% of 1RM	3	16.6	147 ± 9.4	138.8 ± 8.4	− 5.91	95.8 ± 7.9	89.8 ± 8	− 6.68
Heffernan, 2013^28^	11	61 ± 1	M/F	Not	50% of 1RM	3	12	134 ± 5	129 ± 4	− 3.88	84 ± 2	77 ± 2	− 9.09
Moeini, 2015^23^	20	57.5 ± 8.6	M /F	Yes (unspecified)	?	2	8	128.21 ± 15.4	116.42 ± 7.2	− 10.13	82.50 ± 9.4	81.78 ± 8.0	− 0.88
Moraes, 2012^33^	15	46 ± 3	M	Not	60% of 1RM	3	12	150 ± 3	134 ± 3	− 11.94	93 ± 2	81 ± 1	− 14.81
Moreira, 2014^29^	20	66.8 ± 5.6	F	Yes (beta-Blocker; Calcium channel blockers; ACEI; Diuretic)	70% of 1RM	3	12	125.2 ± 9.3	114.7 ± 9.8	− 9.15	72.0 ± 6.8	71 ± 5.7	− 1.41
Mota, 2013^20^	32	67.5 ± 7.0	F	Yes (Statins; ACE inhibitors, Diuretics)	70% of 1RM	3	16	134.5 ± 14.6	120.2 ± 11.8	− 11.90	76.0 ± 9.2	72.4 ± 9.3	− 4.97
Nascimento, 2014^31^	12	67.6 ± 6.4	F	Yes (Statins; ACE inhibitors, diuretics, calcium channel blockers, and many others)	Moderate intensity of Borg scale	2	14	130.60 ± 8.1	112.50 ± 9.7	− 16.09	80.60 ± 7.6	70.50 ± 9.5	− 14.33
Nascimento, 2018^6^	14	68.5 ± 6.4	F	Yes (angiotensin receptor blocker; diuretics, b-blockers, and many others)	10RM test	2	10	121.60 ± 10.4	113,77 ± 10.8	− 6.88	71.37 ± 5.5	70.51 ± 6.0	− 1.22
Tomeleri, 2017^32^	15	69 ± 6.6	F	Yes (beta-blockers; calcium channel blockers and ACE-inhibitors/Angiotensin II-antagonists)	10-15RM test	2	12	142.2 ± 10.5	130.1 ± 9.7	− 9.30	79.5 ± 7.0	72.8 ± 4.3	− 9.20

*RM* repetition maximum, *ACE* Angiotensin-converting enzyme, *ACEI* Angiotensin-converting enzyme inhibitor, *Δ %* variation of arterial hypertension. Age and Blood Pressure values were presented with mean ± SD (standard deviation). *M* male, *F* female. Unspecified = does not say which drug was used.

### Primary outcome

**Figure 2:**
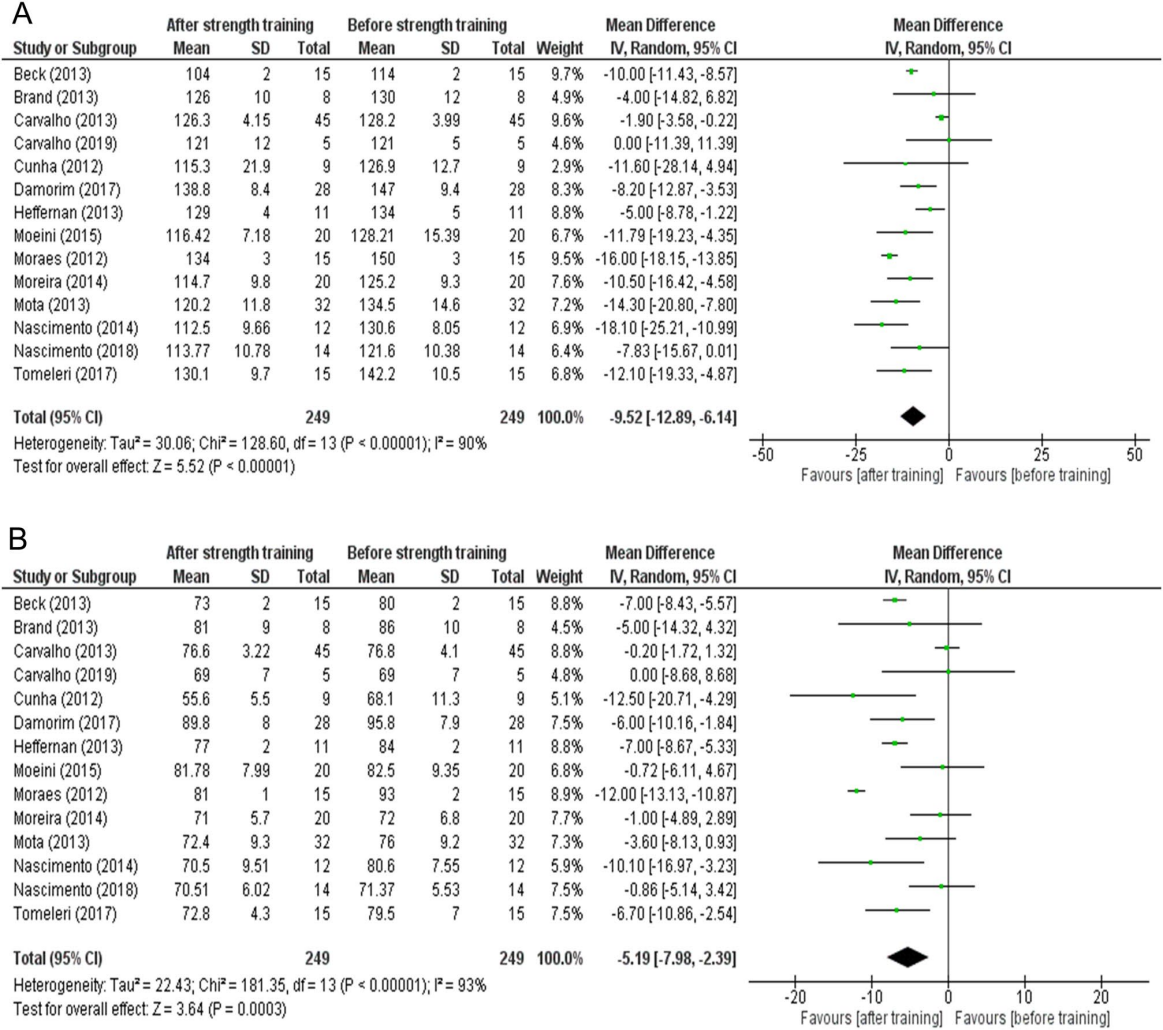
(**A**) Effects of strength training on systolic blood pressure in overall hypertensive individuals. (**B**) Effects of strength training on diastolic blood pressure in general hypertensive individuals.

Figure 2A,B show the overall estimates of the random-effects meta-analysis of studies with baseline and post-training hypertension responses. When comparing hypertension, the results of systolic blood pressure after strength training were significantly decreased by strength training compared to the baseline moment (mean difference = − 9.52; 95% CI − 12.89 to − 6.14; I^2^ = 90%; p < 0.00001, Fig. 2A). Figure 2B shows significant associations between diastolic blood pressure and strength training (mean difference = − 5.19; 95% CI − 7.98 to − 2.39; I^2^ = 93%; p = 0.0003).

### Secondary outcomes

In an attempt to identify the most effective prescription of the training variables that make up the training volume, the term ‘load intensity’ was used when training was prescribed based on a load (e.g., 50% or 70% of the 1RM) and the term intensity was used when training was prescribed based on cardiovascular parameters (40% of maximum heart rate), considering the concept that intensity is the level of effort applied to a given load^34,35^. However, in the current review, the term load intensity will be displayed at all times.

#### Age

**Figure 3:**
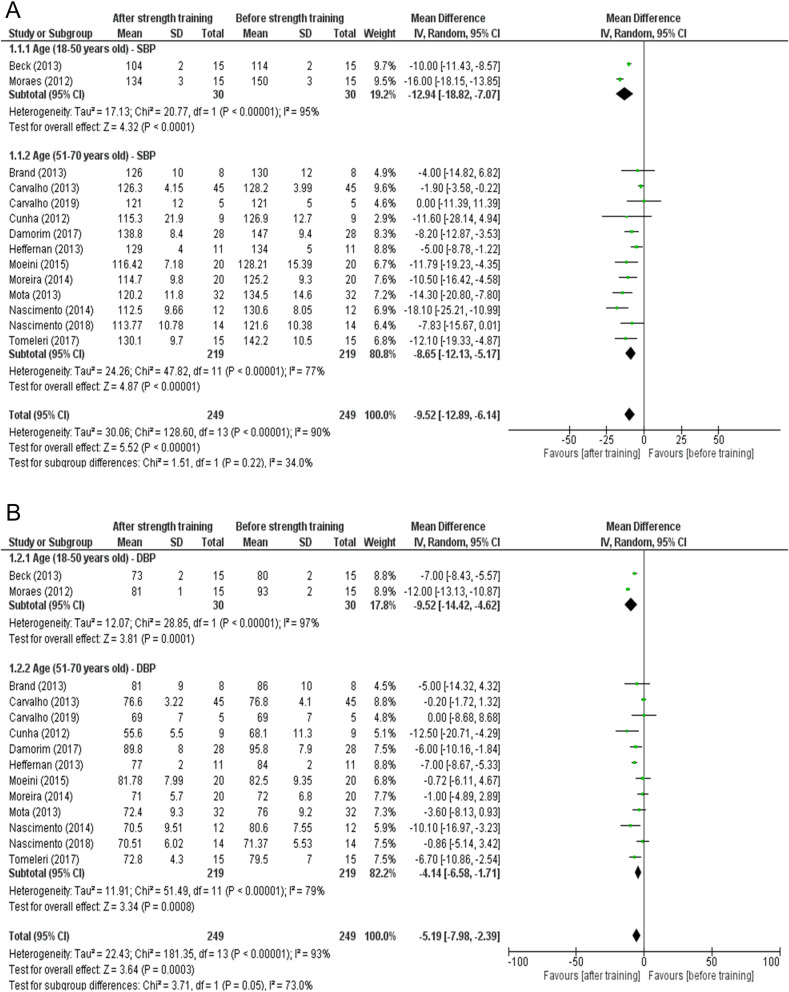
(**A**) Effects of strength training on systolic blood pressure in hypertensive individuals aged 18–50 and 51–70 years old. (**B**) Effects of strength training on diastolic blood pressure in hypertensive individuals with 18–50 and 51–70 years.

We performed a subgroup comparison of participants aged 18–50 years, and found a difference in SBP before and after strength training (mean difference = − 12.94; 95% CI − 18.82 to − 7.07; I^2^ = 95%; p < 0.0001; Fig. 3A). The comparative analysis between participants aged 51–70 years showed a significant reduction in SBP after strength training compared to before strength training (mean difference = − 8.65; 95% CI − 12.13 to − 5.17; I^2^ = 77%; p < 0.00001; subgroup difference = I^2^ = 34%; p = 0.22; Fig. 3A).

The results of diastolic pressure were significant in both subgroups. Among participants aged 18–50 years, the after-strength training blood pressure values were lower compared to baseline (mean difference = − 9.52; 95% CI − 14.42 to − 4.62; I^2^ = 97%; p = 0.0001; Fig. 3B). Comparing study participants aged between 51 and 70 years, we observed a significant reduction in DBP after strength training when compared to before training (mean difference = − 4.14; 95% CI − 6.58 to − 1.71; I^2^ = 79%; p = 0.0008; subgroup difference = I^2^ = 73%; p = 0.05; Fig. 3B).

#### Load intensity

**Figure 4:**
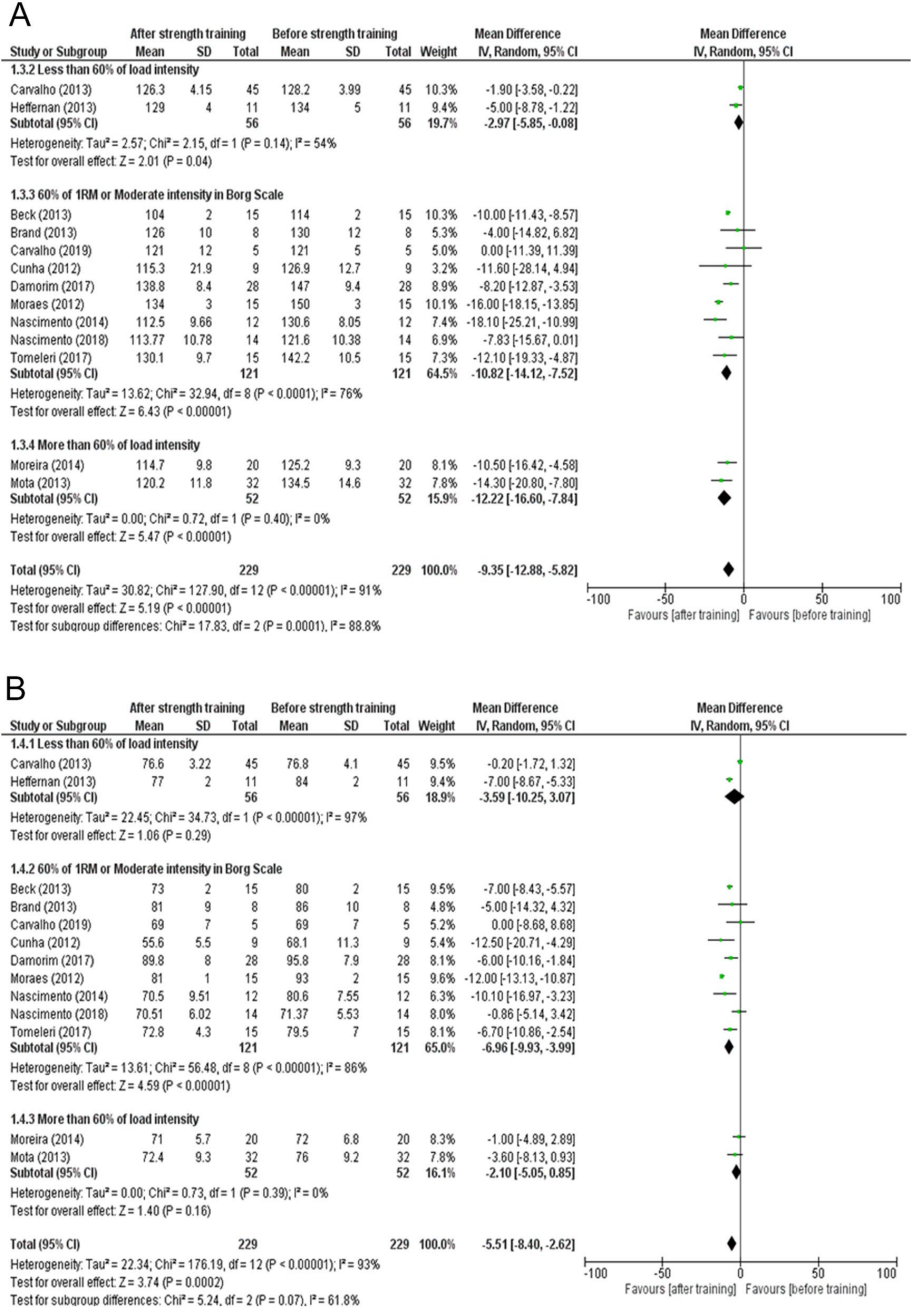
(**A**) Effects of strength training on systolic blood pressure in hypertensive individuals with less than 60% load intensity, 60% moderate Borg scale, and more than 60% of load intensity. (**B**) Effects of strength training on diastolic blood pressure in hypertensive individuals with less than 60% of load intensity, 60% moderate Borg scale, and more than 60% of load intensity.

Most of the included studies used a load intensity of 60%^25,26,33^ or 70%^20,29^ of the 1RM. Other studies used the Borg scale with intensity scoring of 11–15^27^ and 11–13 points^24^, besides also using 40–60% of the heart rate^22^, 50%^28^ of the 1RM, the 10RM test^6^, and the 10-15RM test^32^. One study did not indicate the intensity and was excluded^23^. These parameters indicated the predominant moderate load intensity of the strength training protocols (Fig. 4A indicates data on SBP, and Fig. 4B indicates data on DBP). This meta-analysis shows that the most commonly used load intensity in strength training for the treatment of arterial hypertension is 60–70% of 1RM, and that studies which used a load intensity of more than 60% are considered more homogeneous (I^2^ = 0%—both SBP and DBP). In addition, we can see a robust reduction in AH values accompanied by a statistically significant change in SBP (mean difference = − 12.22; 95% CI − 16.60 to − 7.84; p-value < 0.00001, Fig. 4A) and DBP (mean difference = − 2.10; 95% CI − 5.05 to 0.85; p-value = 0.16, Fig. 4B). A load intensity of less than 60%, even with high heterogeneity, led to a significant reduction in systolic blood pressure (mean difference = − 2.97; 95% CI − 5.85 to − 0.08; I^2^ = 54%; p-value = 0.04, Fig. 4A), but not in diastolic blood pressure (mean difference = − 3.59; 95% CI − 10.25 to 3.07; I^2^ = 97%; p-value = 0.29, Fig. 4B). A moderate load intensity showed high heterogeneity, but a significant reduction in blood pressure values in hypertensive individuals for both systolic (mean difference = − 10.82; 95% CI − 14.12 to − 7.52; I^2^ = 76%; p-value < 0.00001, Fig. 4A) and diastolic pressure (mean difference = − 6.96; 95% CI − 9.93 to − 3.99; I^2^ = 86%; p-value < 0.00001, Fig. 4B). All studies used resistance machines. Moeini et al.^23^, also supplemented with free weights. All exercises were performed dynamically.

#### Frequency and duration

**Figure 5:**
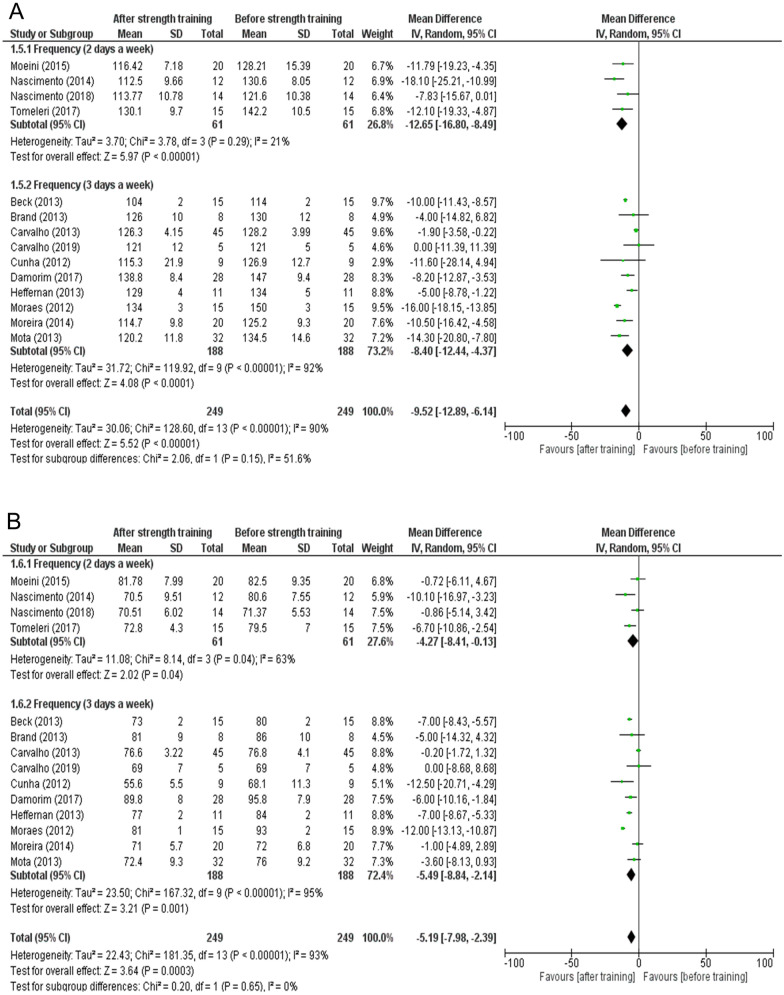
(**A**) Effects of strength training on systolic blood pressure in hypertensive individuals with 2 and 3 days a week of training frequency. (**B**) Effects of strength training on diastolic blood pressure in hypertensive individuals with 2 and 3 days a week of training frequency.

The forest plot of the change in blood pressure according to the weekly frequency of the strength training protocols is presented in Fig. 5. The majority of the included studies used a training frequency of 3 days per week^20,22,24–30,33^, while few studies used a training frequency of 2 days per week^6,23,31,32^. Lower heterogeneity was observed in the 2 days a week subgroup (I^2^ = 21%, Fig. 5A), and this was accompanied by a significant difference (mean difference = − 12.65; 95% CI − 16.80 to − 8.49; p-value < 0.00001, Fig. 5A) in SBP (subgroup difference = I^2^ = 51.6%; p-value = 0.15, Fig. 5A). Furthermore, the 2 days a week subgroup presented greater homogeneity (I^2^ = 63%, Fig. 5B) and statistically significant difference (mean difference = − 4.27; 95% CI − 8.41 to − 0.13; p-value = 0.04, Fig. 5B) in DBP (subgroup difference = I^2^ = 0%; p-value = 0.65, Fig. 5B).

**Figure 6:**
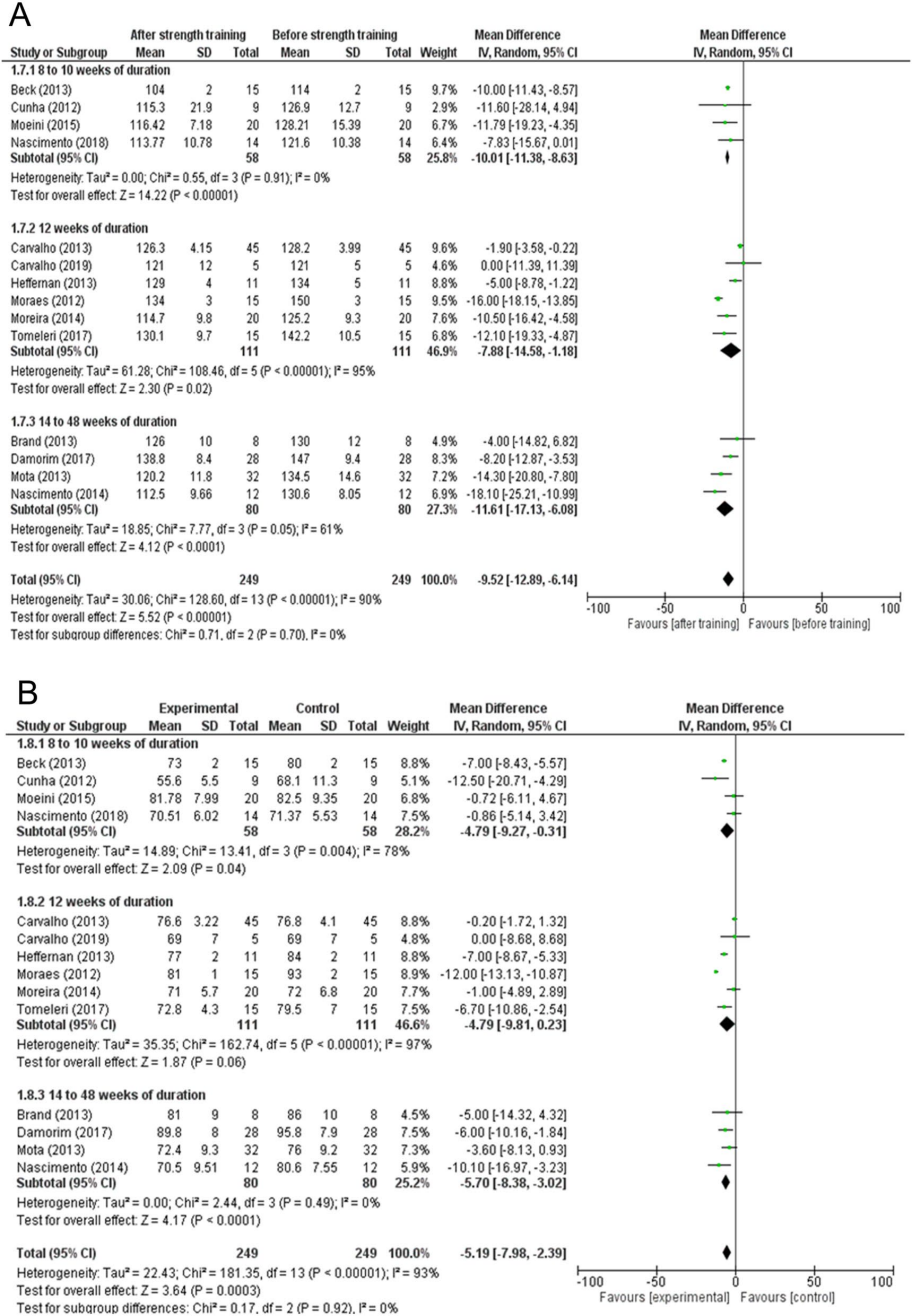
(**A**) Effects of strength training on systolic blood pressure in hypertensive individuals with 8 to 10, 12, and 14 to 48 weeks of training duration. (**B**) Effects of strength training on diastolic blood pressure in hypertensive individuals with 8 to 10, 12, and 14 to 48 weeks of training duration.

The forest plot of the change in blood pressure based on the duration of the intervention is presented in Fig. 6. The study with the longest period of intervention was Brand et al.^27^, with 48 weeks of strength training. The most common period of intervention was 12 weeks of strength training^22,24,28,29,32,33^. In addition, other periods were used, such as 16.6 weeks^25^, 8 weeks^23,26,30^, 10 weeks^6^, 14 weeks^31^, and 16 weeks^20^. This meta-analysis showed that all subgroups of duration of strength training were efficient for the reduction in blood pressure in hypertensive individuals. However, the 8–10 weeks subgroup showed greater homogeneity (I^2^ = 0%, Fig. 6A), and this was accompanied by a statistically significant change (mean difference = − 10.01; 95% CI − 11.38 to − 8.63; p-value < 0.00001; subgroup difference = I^2^ = 0%; p-value = 0.70, Fig. 6A) in SBP. On the other hand, for DBP, the 14–48 weeks subgroups showed greater homogeneity (I^2^ = 0%, Fig. 6B) values, and this was accompanied by a statistically significant change (mean difference = − 5.70; 95% CI − 8.38 to − 3.02; p-value < 0.00001; subgroup difference = I^2^ = 0%; p-value = 0.92, Fig. 6B). Studies that used long periods of strength training, such as 14–48 weeks, even with less homogeneity (I^2^ = 61%) showed a greater SBP reduction in relation to the other groups, with a shorter physical training protocol (mean difference = − 11.61; 95% CI − 17.13 to − 6.08; p value < 0.00001, Fig. 6A). This leads to the hypothesis that an effect on arterial hypertension is dependent on the duration of strength training.

#### Description of study quality

**Figure 7:**
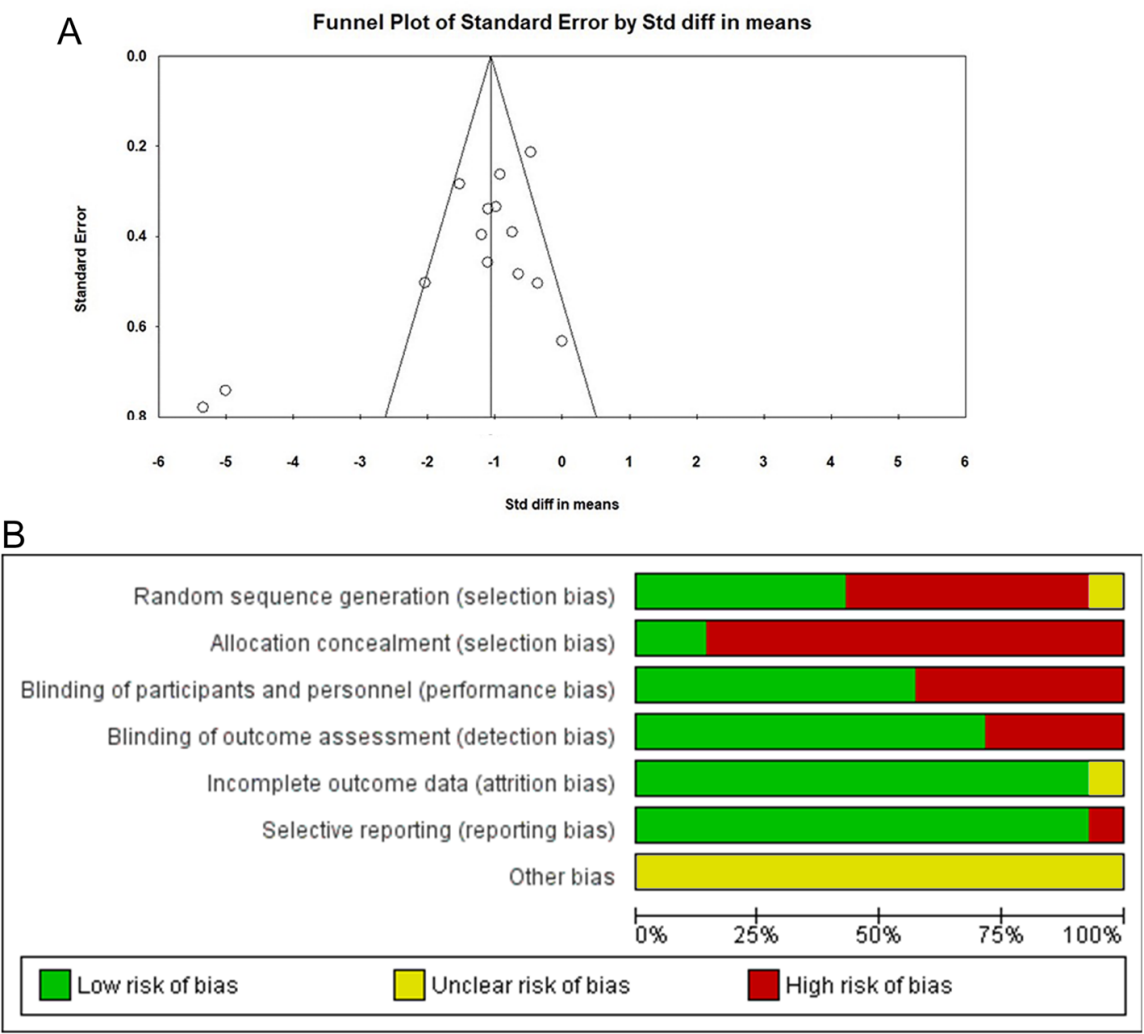
(**A**) Funnel plot for the meta-analysis of strength training such as treatment of hypertension. Egger’s test (P = 0.01729) shows a significant publication bias. Beck et al.^26^ and Moraes et al.^33^ are the biggest outliers. (**B**) Risk of bias graph.

Details of the risk of bias assessment are elucidated in Fig. 7B. None of the studies received a high risk of bias in all categories. The studies included in this review relate to heterogeneous patients. The funnel plot shows the asymmetric distribution, suggesting the presence of publication bias (Fig. 7A).

## Discussion

SAH affects a large part of the population, being one of the most common cardiovascular diseases, and can lead to left ventricular hypertrophy (LVH) and heart failure^36^. Changes in individual lifestyles, such as an increase in physical activity level, emerge as an important non-pharmacologic treatment for hypertension^37^, as it promotes chronic and acute adaptations in the cardiovascular system, decreasing heart rate and blood pressure in hypertensive individuals^38,39^, and adaptations in cardiac function^40^.

This meta-analysis and systematic review of randomized clinical trials involving 253 participants aimed to analyze the breadth of evidence on the treatment potential of strength training in adults and older people with hypertension and to verify the intensity, volume, and duration of training with greater effects. The analyses showed that strength training can significantly improve arterial hypertension. In addition, we identified age and strength training variables that may partially modify the effects of strength training on hypertension.

The current meta-analysis shows that strength training interventions significantly reduced SBP and DBP in hypertensive participants when compared to baseline. The decrease in AH values was greater in SBP when compared to DBP, indicating hemodynamic exercise adaptation in heart systolic movement. The only study that did not show a decrease in blood pressure values from strength training in hypertensive individuals was by Carvalho et al.^24^, but these data may be due to the assessment of blood pressure, which was performed just 24 h after the training. With these results, we observed that the SBP showed greater sensitivity to strength training compared to the DBP. Although the exact biological mechanisms are not clear, it is possible to identify that strength training was effective for the cardiovascular health of the participants.

The mechanisms of decreased blood pressure through aerobic training have been well studied^14,41^. However, there have been few investigations on strength training. One of the hypotheses for this result would also be that the increase in NO synthesis with strength training causes vasodilation^32^. Another hypothesis considers the decrease in sympathetic discharge in the post-exercise period^42,43^. Studies using strength training in hypertensive patients have reported a reduction in adrenaline levels^44^, and blood glucose and LDL levels^24^. However, these studies used different training protocols, with varied load intensity. Hypertension conditions increase the levels of circulating Angiotensin-II (Ang-II)^45^ and Endothelin-1 (ET-1)^46^, powerful vasoconstrictors^45,47,48^. However, even with lower blood pressure levels, strength exercise is less efficient in reducing ET-1 in hypertensive individuals^41,49^. This suggests that decreases in arterial pressure can be mediated by other metabolites, such as cytokines and/or NO. It was shown that 12 weeks of strength exercise can significantly decrease levels of NO metabolites in hypertensive women, and this was positively correlated with a decrease in systolic blood pressure^32^. With this, studies that explore NO as a possible mediator of the reduction in arterial hypertension in hypertensive individuals from strength training are suggested.

Hypertension is a multifactor disease that progresses with age and has a greater prevalence in older adults^50^. In this meta-analysis, we also found that the size of the effect of the strength training intervention can be affected by the age of the participant. Hypertensive individuals aged 18–50 years showed considerably greater hypotensive effects promoted by strength training compared to individuals aged 51–70 years. The aging process is associated with lower NO production, oxidative stress, and endothelial dysfunction^51^, and this is exacerbated by the inflammatory state caused by hypertension^50^. On the other hand, strength exercise is involved in increasing NO and controlling inflammation^32,52^. These age changes may explain the lower, but significant, hypotensive response of the 51- to 70-year-old group. Our studies support the idea that strength training can be performed at any age, as even in older people there are hypotensive benefits of physical strength training.

Among the objectives proposed in this review was confirmation of the effects of strength training for arterial hypertension, identifying the number of necessary sessions, load intensity, and volume for the treatment of hypertension through strength training. The articles collected through the database search gathered studies that analyzed the effects of strength training in hypertensive patients. The results of the reviewed studies suggest that strength training with moderate to vigorous load intensity has a positive effect on reducing systolic and diastolic blood pressure, which confirms its recommendation as a treatment for arterial hypertension^53^.

This study found differences in the interventions applied to strength training based on the applied protocols and showed results around the 20th training session, in comparison to the hypotensive data of the aerobic physical training, which showed results around the 10th session of physical training^25^. We also verified that the hypotensive effects of strength training are effective for about 14 weeks after detraining^31^, different from the effects promoted by aerobic physical exercise^54^. These findings support the important role of strength training in reducing mortality risk, especially for cardiovascular diseases^55^. Future studies should focus on cellular and molecular mechanisms responsible for this decrease in blood pressure values through strength training.

When training volume is equalized, there are no significant differences in muscle adaptations with lower or higher training frequency in trained^56^ and untrained individuals^57^, indicating that strength training volume is the main training variable. In normotensive individuals, moderate and vigorous load intensity is related to blood pressure reduction^58^, when different weekly frequencies are not related^59^. In our review, we found a dose–response relationship between load intensity and duration on SBP, but this relationship was not observed in the weekly frequency variable. This has also been reported in another review^9^. These findings corroborate the concept that the volume of strength training is more important than the weekly frequency for the reduction in blood pressure in hypertensive individuals.

This review has several limitations. First, we did not exclude studies that made use of anti-hypertensive drugs, so, this must be considered when interpreting the results. Second, the included articles used different types of control groups, among them hypertensive individuals not submitted to exercise intervention; normotensive with exercise intervention; normotensive without exercise intervention. However, only the values of hypertensive individuals were computed. Third, some studies utilized men and women in the same intervention group, which prevents sensitivity analysis of the effects of strength training according to sex.

## Conclusion

The present data suggest that strength training, performed with a moderate to vigorous load intensity, 2 or 3 days a week, performed for at least 8 weeks, is a good strategy to decrease blood pressure in hypertensive individuals.

## Methods

We conducted the present meta-analysis following the protocol that was previously registered in the PROSPERO database (CRD42020151269), and the protocol has previously been published^60^. The protocol for this systematic review and meta-analysis, as previously established, followed the checklist of the Cochrane Handbook for Systematic Reviews of Interventions and the Preferred Reporting Items for Systematic Reviews^60^.

### Eligibility criteria

To select studies that could answer our research objectives, we used the following criteria: (I) studies that used an intervention with strength training to control arterial hypertension; (II) studies that used strength training performed for at least 8 weeks; (III) we selected studies with a control group, aerobic exercise group as a comparison; (IV) studies with a methodology using blood pressure monitoring at least during the initial period and after the intervention period.

This systematic review based on the previously published protocol^60^ included studies analyzing strength training in male and female patients with established hypertension. Studies with interventions such as sports, other types of exercises, such as Pilates, stretching, yoga, or physical activities that did not have adequate specifications of exercises used in the methodology were not considered for analysis. Furthermore, we excluded nonrandomized and crossover trials, studies without intervention, case studies, and meta-analyses from this systematic review. We used studies in English and Portuguese in the search.

Based on the designed protocol^60^, we included randomized clinical trials that analyzed the effect of strength training on participants with SAH, developed between 2009 and December 2020.

We used the PICOS strategy as an eligibility criterion as stated in the published protocol^60^.

#### Population

Studies including adult participants of 18 years and above (without age limitation), of both sexes, diagnosed with pre-hypertension and/or hypertension.

#### Intervention

Randomized control trials with strength exercise interventions at least 2 times a week. Interventions that included more than one type of exercise were excluded, and studies that performed combined strength training simultaneously with any other type of multimodal physical exercise were excluded from this review. Studies evaluating only normotensive individuals were excluded.

#### Comparison

Studies with both groups (hypertensive and normotensive) were included, but only the values of the hypertensive group were considered in the compilation of the results. Although studies with interventions in strength training and aerobic training were included, only information about strength training was considered in the compilation of the results. The region, nation, and ethnic origin were not limited in this review.

#### Outcomes

This review aimed to analyze the breadth of evidence on the therapeutic potential of strength training in arterial hypertension. We used studies that considered interventions such as resistance training and strength training. Studies that performed isometric and dynamic strength training interventions were included. The outcome of this systematic review seeks to describe strength training interventions that were effective in improving blood pressure in hypertensive adults, as well as establishing which of the strength training protocols used were more efficient in reducing systolic and diastolic blood pressure, seeking to discuss, synthesize, and determine the most efficient duration of intervention and type of protocol to use for significant effects on blood pressure.

#### Types of study

RCTs comparing hypertensive patients with a pre- and post-strength training intervention of at least 8 weeks were included.

### Date search and study identification

As proposed in protocol^60^, all studies using strength training as an intervention to treat arterial hypertension in men or women published between 2009 and December 2020 were included in this search. The search was performed by two independent raters (RRC and JCR). The databases used for electronic research were MEDLINE (through PubMed) and the Lilacs Cochrane Library. The keyword combination is mentioned in the protocol, summarizing strength training, blood pressure, and prehypertensive and hypertensive participants. We used the title and abstract for the selection of all studies (the filters are in the supplementary material).

### Data extraction and analysis

Studies indicated by the electronic search strategy^60^ were exported to a database configured by EndNote software, where duplicates were removed and the following items were extracted; (A) general information: authors, the title of the article, journal, year of publication, contact of the corresponding author, affiliation of authors; (B) methodology: RCT design, number of participants, age, sex, and medication use; (C) Intervention: strength training, load intensity, weekly volume, and duration of the intervention; (D) Result: pre-intervention blood pressure, post-intervention blood pressure; (E) Other information: Ethics Committee approval number, financial support, conflict of interest, and a complete list of references.

Two reviewers (RRC and JCR) evaluated eligibility based on inclusion and exclusion criteria and independently selected studies by title and abstract. In case of doubts, the full text was read and discussed among the reviewers to verify if the study met the criteria proposed by this systematic review. A third reviewer was called when there was a conflict between reviewers for the selection and classification of studies. Flow chart A with the summary of the studies analyzed in this systematic review can be found in Fig. 1.

A meta-analysis was performed including studies that met the following criteria: (I) contain strength training interventions, comparators, and their comparable results that can be pooled meaningfully; (II) correct data available, such as mean and standard deviation; or that could be calculated from the data provided by the authors; (III) studies considered sufficiently similar, not showing substantial heterogeneity (above 50%). We used a p-value < 0.1 or I^2^ > 50% to suggest the presence of statistically significant heterogeneity^61^.

The data synthesis and analysis were performed using Review Manager 5.3 software, provided by the Cochrane Collaboration. The Q test results showed either a fixed-effects or random-effects model. The random effects model was used and statistical significance (p < 0.1) was found. A subgroup analysis was performed.

### Risk of bias

To assess the methodological quality of the selected studies, the risk of bias was independently assessed by two reviewers, according to the Cochrane checklist^62^. The following domains were evaluated: (A) Selection bias: random sequence generation and allocation concealment. (B) Performance bias: blinding of participants, investigators, and outcome assessors. (C) Detection bias: blinding of outcome assessment. (D) Attrition bias: incomplete outcome data. (E) Reporting bias: selective outcome reporting. Conflicting scores were discussed with a third reviewer to reach a decision. (F) Other bias: conflicts of interest, follow-up, non-intention-to-treat, or per-protocol analysis. The risk of bias for each selected study was scored independently by each reviewer and was classified as low, high, or uncertain risk of bias.

### Assessment of publication bias

Funnel charts were used to assess publication bias and present the results for the meta-analysis with sufficient articles. We included all eligible data, regardless of methodological quality, and the interpretation of the results was performed based on the asymmetry of the funnel plot. Egger’s test^63^ was conducted using Comprehensive Meta-Analysis V3 software^64^.

### Ethical approval

Data from the participants included in this study were collected from previous peer-reviewed publications, as well as with the approval from the respective research ethics committees.

## Supplementary Information

Supplementary Information 1.

Supplementary Information 2.

Supplementary Information 3.

## Data Availability

The data sets used and/or analyzed during the current study are available from the corresponding author upon reasonable request.
