# Supplementary material for: Strength training for arterial hypertension treatment: a systematic review and meta-analysis of randomized clinical trials
Source: Sci Rep. 2023 Jan 5;13:201. doi: 10.1038/s41598-022-26583-3 (PMC9814600; doi:10.1038/s41598-022-26583-3)
Supplement: Supplementary file 2 — Supplementary Information 2. [file 41598_2022_26583_MOESM2_ESM.pdf]

SUPPLEMENTARY MATERIAL

MEDLINE

Number of localized studies: 4.615

	**Descriptors**	Number of studies reached
**#1**	("Arterial Hypertension") AND (“Resistance Training”)	814
**#2**	("Arterial Hypertension") AND (“*Physical Exercises*”)	3531
**#3**	("Arterial Hypertension") AND (“*Strength Training”*)	270

LILACS (English)

Number of localized studies: 515

	**Descriptors**	Number of studies reached
**#1**	("Arterial Hypertension") AND (“Resistance Training”)	93
**#2**	("Arterial Hypertension") AND (“*Physical Exercises*”)	375
**#3**	("Arterial Hypertension") AND (“*Strength Training”*)	47

PUBMED (English)

Number of localized studies: 515

	**Descriptors**	Number of studies reached
**#1**	("Arterial Hypertension") AND (“Resistance Training”)	659
**#2**	("Arterial Hypertension") AND (“*Physical Exercises*”)	18.943
**#3**	("Arterial Hypertension") AND (“*Strength Training”*)	821

	**Descriptors**	Number of studies reached
**#1**	("hypertension"[MeSh] OR "hypertensive”[TIAB] OR "arterial	602.751

	hypertension"[TIAB]) OR ("blood pressure"[MeSh] OR “arterial pressure”[MeSh] OR "diastolic blood pressure"[TIAB] OR "systolic blood pressure"[TIAB] OR "high blood pressure"[MeSh])	
**#2**	"physical exercise"[MeSh] OR "physical activity"[TIAB] OR "physical training"[TIAB]) OR ("exercise training"[MeSh] OR "strength training"[TIAB] OR "force exercise"[TIAB])	146.024
**#3**	("strength exercise"[MeSh] OR "power exercise"[TIAB] OR "resistance exercise"[TIAB])	6.762
**#4**	("physical exercise"[MeSh] OR "physical activity"[TIAB] OR "physical training"[TIAB]) OR ("exercise training"[MeSh] OR "strength training"[TIAB] OR "force exercise"[TIAB]) NOT (“aerobic physical” [MeSh] OR “aerobic training” [MeSh] OR “physical fitness” [TIAB])	142.172
**#5**	(“#1” AND “#2”AND “#3”)	75
**#6**	(“#1” AND “#2”AND “#4”)	8.917
