# Supplementary material for: Strength training for arterial hypertension treatment: a systematic review and meta-analysis of randomized clinical trials
Source: Sci Rep. 2023 Jan 5;13:201. doi: 10.1038/s41598-022-26583-3 (PMC9814600; doi:10.1038/s41598-022-26583-3)
Supplement: Supplementary file 1 — Supplementary Information 1. [file 41598_2022_26583_MOESM1_ESM.zip › Model_search.doc]

**SUPPLEMENTARY MATERIAL**

**MEDLINE**

**Number of localized studies:** 4.615

**Limits:** XXX

**Number of studies after applying limits:** 33

|  | **Descriptors** | Number of studies reached |
| --- | --- | --- |
| **#1** | ("Arterial Hypertension") AND (“Resistance Training”) | 814 |
| **#2** | ("Arterial Hypertension") AND (“*Physical Exercises*”) | 3531 |
| **#3** | ("Arterial Hypertension") AND (“*Strength Training”*) | 270 |

**LILACS (English)**

**Number of localized studies**: 515

**Limits:** *XXX*

**Number of studies after applying limits:** 24

|  | **Descriptors** | Number of studies reached |
| --- | --- | --- |
| **#1** | ("Arterial Hypertension") AND (“Resistance Training”) | 93 |
| **#2** | ("Arterial Hypertension") AND (“*Physical Exercises*”) | 375 |
| **#3** | ("Arterial Hypertension") AND (“*Strength Training”*) | 47 |

**PUBMED (English)**

**Number of localized studies**: 515

**Limits:** *XXX*

**Number of studies after applying limits:** 24

|  | **Descriptors** | Number of studies reached |
| --- | --- | --- |
| **#1** | ("Arterial Hypertension") AND (“Resistance Training”) | 659 |
| **#2** | ("Arterial Hypertension") AND (“*Physical Exercises*”) | 18.943 |
| **#3** | ("Arterial Hypertension") AND (“*Strength Training”*) | 821 |

|  | **Descriptors** | Number of studies reached |
| --- | --- | --- |
| **#1** | ("hypertension"[MeSh] OR "hypertensive”[TIAB] OR "arterial hypertension"[TIAB]) OR ("blood pressure"[MeSh] OR “arterial pressure”[MeSh] OR "diastolic blood pressure"[TIAB] OR "systolic blood pressure"[TIAB] OR "high blood pressure"[MeSh]) | 602.751 |
| **#2** | "physical exercise"[MeSh] OR "physical activity"[TIAB] OR "physical training"[TIAB]) OR ("exercise training"[MeSh] OR "strength training"[TIAB] OR "force exercise"[TIAB]) | 146.024 |
| **#3** | ("strength exercise"[MeSh] OR "power exercise"[TIAB] OR "resistance exercise"[TIAB]) | 6.762 |
| **#4** | ("physical exercise"[MeSh] OR "physical activity"[TIAB] OR "physical training"[TIAB]) OR ("exercise training"[MeSh] OR "strength training"[TIAB] OR "force exercise"[TIAB]) NOT (“aerobic physical” [MeSh] OR “aerobic training” [MeSh] OR “physical fitness” [TIAB]) | 142.172 |
| **#5** | (“#1” AND “#2”AND “#3”) | 75 |
| **#6** | (“#1” AND “#2”AND “#4”) | 8.917 |
